# Stochastic resonance improves visuomotor temporal integration in healthy young adults

**DOI:** 10.1371/journal.pone.0209382

**Published:** 2018-12-14

**Authors:** Satoshi Nobusako, Michihiro Osumi, Atsushi Matsuo, Takahiro Fukuchi, Akio Nakai, Takuro Zama, Sotaro Shimada, Shu Morioka

**Affiliations:** 1 Neurorehabilitation Research Center, Kio University, Nara, Japan; 2 Graduate School of Health Science, Kio University, Nara, Japan; 3 Department of Physical Therapy, Faculty of Health Sciences, Kio University, Nara, Japan; 4 Graduate School of Clinical Education & The Center for the Study of Child Development, Institute for Education, Mukogawa Women’s University, Hyogo, Japan; 5 Rhythm-Based Brain Information Processing Unit, RIKEN CBS-TOYOTA Collaboration Center, RIKEN Center for Brain Science, Saitama, Japan; 6 Department of Electronics and Bioinformatics School of Science and Technology, Meiji University, Kanagawa, Japan; University of Rome, ITALY

## Abstract

Mechanical and electrical noise stimulation to the body is known to improve the sensorimotor system. This improvement is related to stochastic resonance (SR), a phenomenon described as a “noise benefit” to various sensory and motor systems. The current study investigated the influence of SR on visuomotor temporal integration and hand motor function under delayed visual feedback in healthy young adults. The purpose of this study was to measure the usefulness of SR as a neurorehabilitation device for disorders of visuomotor temporal integration. Thirty healthy volunteers underwent detection tasks and hand motor function tests under delayed visual feedback, with or without SR. Of the 30 participants, 15 carried out the tasks under delayed visual feedback in the order of SR on-condition, off-condition, off-condition, and on-condition. The remaining 15 participants conducted the experimental tasks in the order of SR off-condition, on-condition, on-condition, and off-condition. Comparisons of the delay detection threshold (DDT), steepness of the delay detection probability curves, box and block test (BBT) scores, and nine-hole peg test (NHPT) scores between the SR on- and off-conditions were performed. The DDT under the SR on-condition was significantly shortened compared with the SR off-condition. There was no significant difference between the SR on- and off-conditions for the steepness of the delay detection probability curves, BBT scores, and NHPT scores. SR improved visuomotor temporal integration in healthy young adults, and may therefore improve movement disorders in patients with impaired visuomotor temporal integration. However, because the current results showed that SR did not improve hand motor function under delayed visual feedback, it may not improve motor function when a large distortion of visuomotor temporal integration is present. Further studies are required considering several limitations of the current study, and future clinical trials are necessary to verify the effects of motor training using SR for the treatment of visuomotor temporal integration disorders.

## Introduction

Stochastic resonance (SR) is a phenomenon in which the response of a non-linear system to an input signal benefits from the presence of a particular non-zero level of noise [1–9]. The effect of SR is hypothesized to be due the improvement of signal detection in the presence of noise; feedback controlled system performance has been demonstrated in theory [10] and in many biological systems [6, 7, 11–13]. The SR phenomenon provides a pseudo bell-shaped performance curve with a peak in performance at some optimal noise level associated with optimal system output [7]. SR can therefore provide “noise benefits” to some sensory and motor systems [9].

SR has been demonstrated to act on a variety of sensory systems, typically through the use of psychophysical experiments. The application of SR to visual input has been shown to improve contrast sensitivity and detection, the perception of figures, letter recognition, and depth perception [14–17]. SR has also been identified as an important factor in the cochlear coding strategy and contributes to hearing [18–20]. Furthermore, previous studies revealed that vestibular stimulation with SR enhances balance and stability during walking [21, 22]. SR has also been demonstrated to improve tactile sensitivity in healthy individuals and patients with stroke [23–25].

Previous studies have also shown that adding mechanical or electrical noise to the sensorimotor system during static and dynamic tasks can be beneficial to motor task performance [23, 26–30]. Providing mechanical noise to the feet reduces sway in young and elderly subjects and in patients with diabetes and stroke, and improves gait variability in elderly subjects [31–35]. Mechanical noise applied to the ankle muscles improves the balance of patients with functional ankle instability [36]. Electrical noise applied to the tibial nerve improves tactile perception of the soles of the feet in older adults, and electrical noise applied to the back of the knee improves balance [37, 38]. In addition, the application of whole body vibration is an effective treatment to improve the postural stability of patients with Parkinson’s disease [39].

Several studies have also applied SR to improve hand sensation and upper limb-hand movements (such as manual dexterity). Subthreshold vibrotactile noise applied directly to the tip of the index finger has been shown to improve fingertip tactile sensation immediately in patients with stroke [40] and healthy adults [41]. In addition, in healthy adults, SR reduces the amount of extra grip force needed to lift an object, resulting in a more efficient grip [41]. Seo et al. [42] showed that SR immediately improved hand motor function, as measured by the box and block test (BBT) and the nine hole peg test (NHPT), in the paralyzed limbs of hemiplegic patients after stroke.

On the other hand, visuomotor temporal integration is an important function for hand motor control [43–47]. More specifically, it is largely supported by a neural mechanism known as the forward model [48, 49]. The forward model provides stability to the motor system by predicting the sensory outcome of movements before actual sensorimotor feedback becomes available, providing a means of rapid online correction [50–54]. When a time mismatch occurs between motor prediction and actual sensory feedback, error signals are generated in order to correct the initial movement plan [50, 51, 55–61]. Importantly, these error signals act as training signals to refine the accuracy of forward models [44]. Therefore, comparing motor signals and visual feedback to generate temporal errors, i.e., visuomotor temporal integration, is important for hand motor control. In fact, in children with a developmental coordination disorder and adults with limb-apraxia after stroke have deficits in visuomotor temporal integration [45, 47]. In addition, the degree of deficiency in visuomotor temporal integration correlates significantly with the severity of the movement disorder [45–47]. These previous studies showed that the impaired ability to compare self-generated movements and visual feedback to generate temporal errors leads to movement disorders. However, it is not clear whether SR is effective in improving visuomotor temporal integration. Therefore, in the current study, the contribution of SR to an improvement of visuomotor temporal integration was measured, and the possible contribution of SR as a new rehabilitation technology for deficits in visuomotor temporal integration was assessed.

Motor tasks under delayed visual feedback show degraded performance due to the distortion of visuomotor temporal integration. By using a video delay device, it is possible to affect motor performance negatively by inserting a delay into the visual feedback for self-generated movements [62–71]. If SR improves visuomotor temporal integration, there is a possibility that motor performance, as measured under delayed visual feedback, could be improved.

One target of SR measured in the current study was the promotion of visuomotor temporal integration. In the delayed visual feedback detection task, it is necessary to detect the delay of visual information for real-time motor signals and somatosensory feedback. There is a possibility that SR could promote the detection of visual feedback delay. Alternatively, SR may make motor signals and somatosensory feedback more accurate. Another target of SR measured in the current study was the improvement of hand movements under delayed visual feedback. As this task had delayed visual feedback, it was difficult for the participants to determine their movements using visual feedback. Therefore, they were required to increase the weighting of somatosensory feedback and increase motor predictions. The promotion of visuomotor integration could improve somatosensory re-weighting and motor predictions [72], which could be facilitated by the use of SR.

Therefore, the current study investigated whether SR improved visuomotor temporal integration and/or hand motor function in healthy volunteers under a visuomotor temporal incongruent condition. The long-term objective of the present study is to examine the possibility of rehabilitation using SR for movement disorder patients with deficits in visuomotor temporal integration [45–47].

## Material and methods

### Ethics approval and consent to participate

The experimental procedures were approved by the local ethics committee of the Graduate School and Faculty of Health Sciences at Kio University (approval number: H27-33). There were no foreseeable risks to the participants. No personal identification information was collected. The participants provided background information and gave written informed consent. The procedures complied with the ethical standards of the 1964 Declaration of Helsinki regarding the treatment of human participants in research.

### Participants

Thirty healthy young adults (mean age ± standard deviation [SD], 21.0 ± 0.8 years; range, 20–22 years; 16 males) enrolled at Kio University participated in the current study. All participants were right-handed according to the Edinburgh Handedness Inventory [73], and none had a previous diagnosis of a developmental disorder or a physical or mental disability. We only included right-handed participants because we considered the possibility that differences in left and right handedness could affect the results.

### Procedures

**Fig 1:**
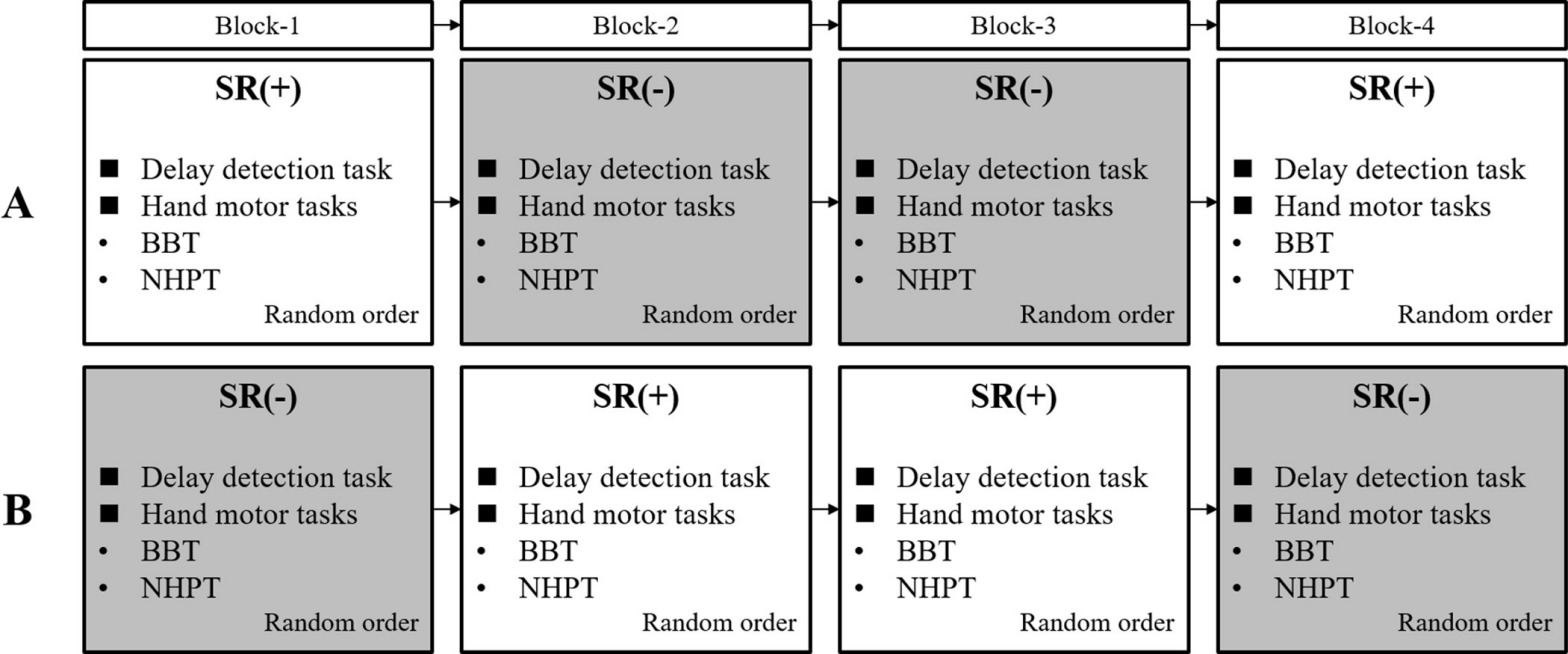
Block design of the experimental protocol. White squares, stochastic resonance (SR) on-condition (+); gray squares, SR off-condition (-); delay detection task, delayed visual feedback detection task; hand motor tasks, hand motor tasks under delayed visual feedback; BBT, box and block test; NHPT, nine hole peg test. (A) Group A: The 15 participants conducted the task in the order of SR on-condition, off-condition, off-condition, and on-condition. (B) Group B: The other 15 participants conducted the task in the order of SR off-condition, on-condition, on-condition, and off-condition. Prior to the start of the four block procedures, the participants received sensory threshold measurements regardless of the SR conditions.

A total of four blocks consisting of two blocks of the SR on-condition and two blocks of the SR off-condition, with the delayed visual feedback detection task and both hand motor tasks under delayed visual feedback, as one set, were used (Fig 1).

Of the 30 participants, 15 (group A; mean age ± SD, 21.0 ± 0.8 years; range, 20–22 years; 8 male participants) carried out the delayed visual feedback detection task and both hand motor tasks under delayed visual feedback, in the order of SR on-condition, off-condition, off-condition, and on-condition (Fig 1A). The remaining 15 participants (group B; mean age ± SD, 21.0 ± 0.8 years; range, 20–22 years; eight male participants), who were the same age and sex as the previous 15 participants, conducted the experimental tasks in the order of SR off-condition, on-condition, on-condition, and off-condition (Fig 1B). These procedures were aimed at offsetting the learning effects of repeated experimental tasks (the delayed visual feedback detection task and both hand motor tasks under delayed visual feedback). The order of the experimental tasks in each block was randomized for each participant.

### SR

Vibrotactile noise was applied using two compact devices (vertical, 10 mm; width, 18 mm; height, 2 mm; Vibration Actuator Sprinter α; Nidec Seimitsu, Santa Clara, CA, USA) attached to the volar and dorsal areas of the wrist of the individual’s right arm using contact tape. The resonance frequency of the device was 170 ± 10 Hz (average ± SD). As in previous studies [25, 42], white noise signals using low-pass filters at 500 Hz were used for the device. White noise signals were outputted from a digital amplifier (FX Audio D802; North Flat Japan, Osaka, Japan) to the SR device (a vibrotactile noise device). Consistent with previous studies [25, 42], we attached the device to the wrist to minimize manual interruption while affecting the tactile sensation of the fingers. The intensity of the vibrotactile noise was set to 60% of the sensory threshold at the start of the test. This intensity has been shown to be the optimum noise level to affect the sensory system [25, 42, 74]. The subjects in the studies of Enders et al. [25] and Seo et al. [42] were elderly stroke survivors, and the subjects in the study of Wells et al. [74] included young adults in their twenties, but the optimum noise level used was the same. Irrespective of whether the SR on- or off-condition was used, sensory thresholds were measured just before starting the four block conditions. To measure the sensory threshold of individual participants, the intensity of the vibrotactile noise was increased gradually, and the level at which the participant perceived noise was taken as the sensory threshold. The vibrotactile noise device was attached at all times during testing and was turned on or off at the beginning of each block according to the SR on-/off-conditions used. The participants were blinded to the noise as they could not feel the noise vibrations.

### Video delay system

**Fig 2:**
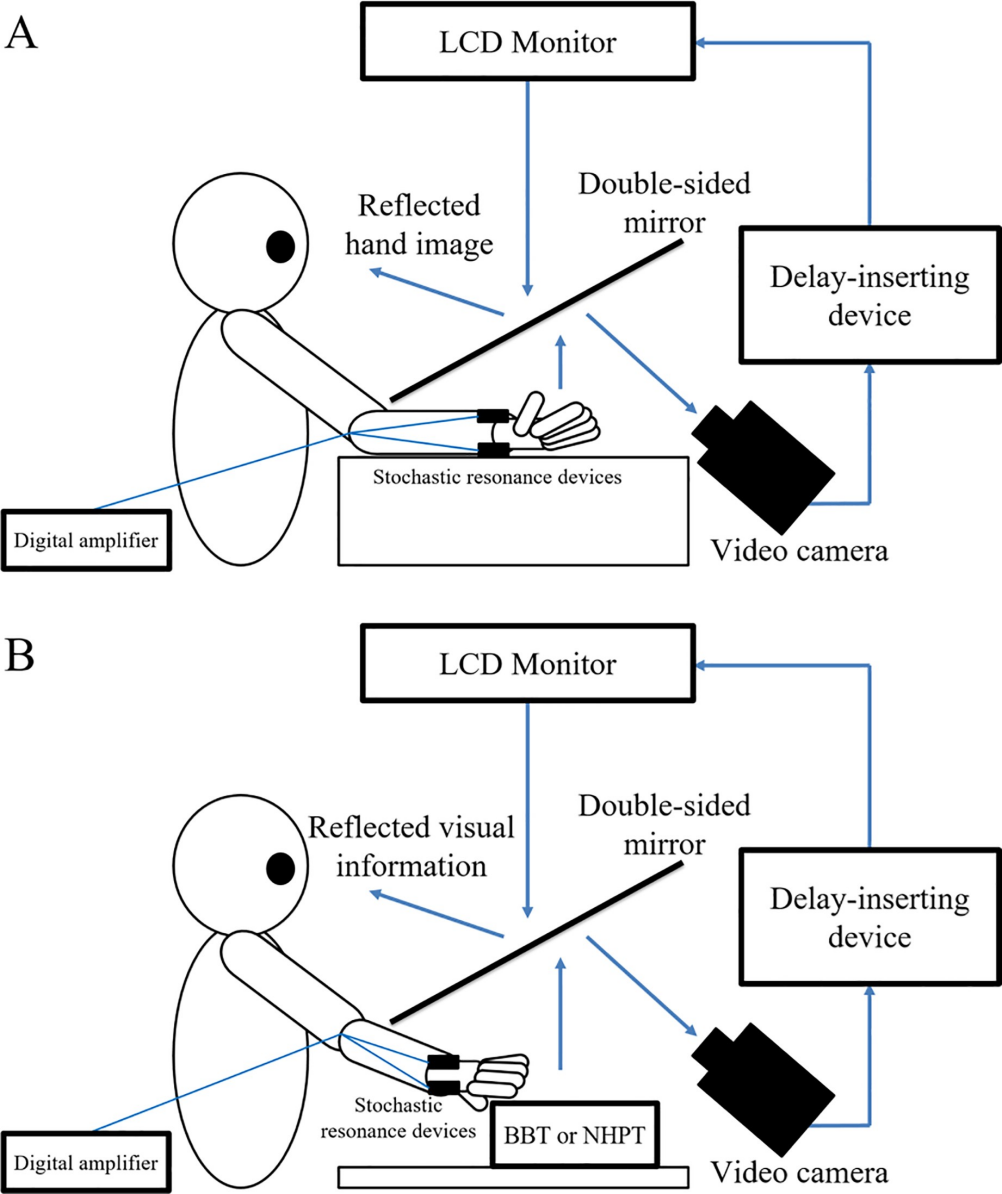
Video delay system and experimental tasks. The video delay system can insert a delay in the visual information from the video camera by the delay-inserting device and then project it to the monitor. Therefore, the participants observed delayed visual feedback with respect to the movement of their hand. (A) Delayed visual feedback detection task. The participants performed the task with an SR device attached to the right wrist. Fifteen delay conditions were set, and the participants were asked to answer whether the visual feedback of their self-generated hand movements was delayed or not delayed. The participants carried out this task in the SR on- and off-conditions. (B) Both hand motor function tests under delayed visual feedback were used. BBT, box and block test; NHPT, nine hole peg test. The participants carried out the BBT and NHPT under a delayed visual feedback of 267 ms with an SR device attached to the right wrist. The participants conducted these tasks in the SR on- and off-conditions.

In this study, an experimental design similar to that of Shimada et al. [75] was used (Fig 2).

A delayed visual feedback detection task and both hand motor tasks under delayed visual feedback were performed using the video delay system. While wearing an SR device on the right wrist, the participant’s right hand was placed under a two-way mirror, and they were unable to see their hand directly. The hand reflected in the two-way mirror was imaged with a video camera (FDR-AXP35; Sony, Tokyo, Japan). The image of the videoed hand was reflected from an installed monitor (LMD-A240; Sony) onto a two-way mirror via a video delay device (EDS-3306; FOR-A YEM Eletex, Tokyo, Japan), and the participant observed the image of their own hand reflected in the mirror. The video delay device created a delayed image captured by the camera at an interval of 33.3 ms, and it was possible to reflect this image from the monitor. Thus, the participant observed the delayed visual feedback of their hand versus the active motion of their hand in real time. In addition, the experimental design included a blackout curtain so the participants were unable to see outside of the experimental chamber. The intrinsic delay of the visual feedback in this experimental setting was 33.71 ms, as measured by a time-lag check device (EDD-5200; FOR-A YEM Eletex).

### Delayed visual feedback detection task

The delayed visual feedback detection task was performed with a vibrotactile noise device attached to the right hand of each participant (Fig 2A). First, the participant received an explanation that the visual feedback of this task had conditions that were not delayed at all and conditions that were delayed by various time intervals. Then, the participant received an explanation that the task had no correct answers and no false answers. In other words, the experimenter explained to each participant that they could answer with free subjective judgment. The participant was instructed to observe the reflection in the mirror with the following instruction: “Please observe your own hand reflected in the mirror.” Subsequently, the participant opened and closed their hand once, in a continuous and smooth manner, according to their own volition, after the experimenter had informed them orally that the trial had started. The self-generated movements were observed under the following 15 delay conditions using a video delay-inserting device: 33, 67, 100, 133, 167, 200, 233, 267, 300, 333, 367, 400, 433, 467, and 500 ms. As with previous studies [45–47, 75–77], this task did not include a 0 ms delay, i.e., no delay condition. During the delayed visual feedback detection task, the participant only looked at the reflection of their hand in the mirror, and not their real hand. Thus, the participant could feel their hand moving while watching the display of the delayed mirror reflection of the same movement. Each participant had to determine if the visual feedback was synchronous or asynchronous relative to the movement of their hand, which was based on their own intention. Immediately following the trial, the participant had to state orally if the visual feedback was “delayed” or “not delayed” by using the forced-choice method. Whether the participant’s report was “delayed” or “not delayed,” the experimenter replied “OK.” In doing so, the experimenter did not give feedback of whether their answers were correct or incorrect. A 10-s rest period was set between each trial. For each block, all 15 delay conditions were treated as one set and were performed five times; their presentation order was randomized. Therefore, each participant completed a total of 75 randomized trials with 15 delay conditions per set of five per block. Furthermore, because there were four blocks in total, with or without SR, a total of 300 randomized tests were completed.

The DDT and the steepness of the probability curve for delay detection, which will be referred to herein as “steepness,” were determined from this task. The DDT was the time delay when the rate of delay detection was 50%; this indicated the extent to which the brain allowed a temporal discrepancy in different modalities of sensation. Steepness indicated the mechanism by which the brain integrated multisensory signals; increased steepness indicated a more strict or precise judgment [75]. Therefore, shortening the DDT and/or increasing steepness represents high visuomotor temporal integration, while prolonging the DDT and/or decreasing steepness represents poor visuomotor temporal integration [45–47, 75]. Previous studies have shown that the DDT is prolonged and steepness is decreased in clinical populations with decreased motor function compared to healthy subjects [45, 47].

A logistic curve was fitted to each participant’s response on the visual feedback delay detection task [45–47, 75, 78], using the formula: P(t) = 1/1+exp(−a(t−DDT)); where t was the visual feedback delay length; P(t) was the probability of delay detection; a was the steepness of the fitted curve; and DDT was the observer’s DDT representing the delay length at which the probability of delay detection was 50%. In the current study, t served as an independent variable, while P(t) was the observed value. The curve was fitted using a nonlinear least squares method (a trust-region algorithm) with the Curve Fitting toolbox in MATLAB R2014b (MathWorks, Inc., Natick, MA, USA) to estimate a and the DDT.

### Hand motor tasks under delayed visual feedback

The participants conducted two-hand motor function tests in the same setup as the delayed visual feedback detection task (Fig 2B). The hand function tests consisted of the BBT and NHPT. These tests provide reliable measurements of manual dexterity [79–81]. Both hand motor function tests were performed consistently under a visual feedback delay of 267 ms using the video delay device. This delay setting was based on the results of several previous studies. Osumi et al. [71] examined the effects of delayed visual feedback on wrist flexion-extension movements using the electromyography activity of the flexor carpi radialis and sense of ownership/heaviness using an experimental setup similar to that used in the current study. Their results showed that a delay of approximately 250 ms reduced the peak frequency of the electromyogram, decreased the sense of ownership, and increased the sensation of heaviness. A delay of approximately 150 ms was not sufficient to affect these parameters. Furthermore, a previous study revealed that the detection threshold of delayed visual feedback of children with a probable developmental coordination disorder was approximately 337 ms, the detection threshold of delayed visual feedback of typical developing children was approximately 247 ms, and the average DDT was approximately 287 ms [45]. In addition, a previous study determined that the DDT of 132 typical developing children from 4 years of age to 15 years of age, was 263.6 ms on average [46]. The average DDT in adult patients with left hemispheric stroke was 238 ms [47]. Therefore, in the current study, we investigated the effect of SR on hand motor function under a delayed visual feedback of 267 ms, in consideration of these previous reports. Therefore, the participants performed both hand motor function tests while observing delayed visual feedback (their own hand and the test objects) of 267 ms against self-generated hand movements. Each hand function test was performed once per block, for a total of four times.

#### BBT

The BBT was carried out according to the implementation procedure and scoring method described by Mathiowetz et al. [82]. The participants carried out this test using the right upper limb of the wrist wearing the vibrotactile noise device. The number of blocks moved from one compartment to the other in 1 min was measured. Therefore, the larger the number of blocks moved, the higher the hand motor function.

#### NHPT

The NHPT was carried out according to the same procedure and scoring method as described by Grice et al. [83]. The participants carried out this test using the right upper limb of the wrist wearing the vibrotactile noise device. The score was the time (in seconds) required to complete the test activity. Therefore, the shorter the time required to complete the test, the higher the hand motor function.

### Statistical analysis

For the comparison of the trial time factors (Block-1-2-3-4, order) of the four indices (DDT, steepness, BBT scores, and NHPT scores) of the two groups (group A, group B), when there was a normal distribution by the Shapiro-Wilk test, a one-factor repeated measures analysis of variance (one-way repeated measures ANOVA) was conducted. When there was no normal distribution, the Friedman test was conducted. For *post hoc* analysis after one-way repeated measures ANOVA, multiple comparisons by the Bonferroni correction were carried out. For *post hoc* analysis after the Friedman test was used, the Wilcoxon signed-rank test was carried out, and the P-value as corrected by the Bonferroni correction was used.

Furthermore, for each participant, the average value of the measurement data in two blocks of the SR on-condition, and the average value of the measurement data in two blocks of the SR off-condition, were calculated and compared. As the DDT and BBT scores had a normal distribution, as determined by the Shapiro-Wilk test, the SR on- and off-conditions were compared using a paired *t*-test. As steepness and the NHPT scores did not have a normal distribution, as determined by the Shapiro-Wilk test, they were compared using the Wilcoxon signed-rank test. In addition, the effect size was calculated [84]. The significance level was set at P < 0.05.

All statistical analyses were performed using SPSS software, version 24 (SPSS, Chicago, IL, USA).

## Results

The raw data of the four indicators of the four blocks in all participants are shown in Table 1.

**Table 1:** Raw data of the four indicators of the four blocks in all participants.

			Block-1				Block-2				Block-3				Block-4			
Group A			SR(+)				SR(-)				SR(-)				SR(+)			
No.	Age (years)	Sex	DDT	Steepness	BBT	NHPT	DDT	Steepness	BBT	NHPT	DDT	Steepness	BBT	NHPT	DDT	Steepness	BBT	NHPT
1	20	male	196.8	0.033	51	62	260.9	0.024	55	45	279.5	0.021	48	48	161.2	0.056	51	46
2	20	female	196.2	0.017	53	112	224.0	0.014	62	67	222.9	0.013	60	58	206.8	0.017	61	59
3	20	male	199.7	0.027	51	59	207.2	0.031	52	47	165.7	0.300	62	53	135.8	0.047	65	49
4	20	female	163.5	0.399	60	58	192.8	0.045	64	54	158.3	0.064	64	48	136.5	0.395	64	43
5	20	male	168.5	0.266	48	63	167.4	0.056	52	51	158.3	0.064	54	47	134.4	0.281	57	47
6	21	female	124.0	0.029	58	54	108.5	0.015	62	52	136.5	0.027	56	41	72.4	0.014	62	47
7	21	male	212.2	0.039	51	68	167.4	0.056	50	64	212.7	0.029	54	50	130.7	0.040	61	51
8	21	female	116.6	0.049	46	40	141.4	0.163	52	44	130.7	0.040	53	62	103.7	0.373	53	52
9	21	male	150.0	0.032	62	86	141.4	0.163	62	48	168.2	0.324	62	63	141.7	0.064	65	40
10	21	female	151.3	0.019	64	84	113.0	0.036	71	57	122.6	0.044	79	44	101.7	0.071	87	33
11	22	male	250.0	0.024	51	56	246.6	0.033	57	45	189.3	0.051	53	40	179.5	0.030	56	42
12	22	female	217.6	0.068	46	56	200.2	0.027	49	59	210.7	0.020	51	46	166.2	0.035	49	44
13	22	male	128.9	0.040	60	44	158.3	0.064	63	37	109.4	0.027	59	60	83.5	0.085	59	55
14	22	female	131.7	0.303	71	55	150.0	0.547	72	62	165.4	0.261	78	39	121.0	0.039	83	47
15	22	male	165.2	0.070	66	51	135.3	0.037	65	53	131.5	0.274	69	49	129.6	0.401	67	48
Mean	21.0		171.5	0.094	55.9	63.2	174.3	0.087	59.2	52.3	170.8	0.104	60.1	49.9	133.6	0.130	62.7	46.9
Standard deviation	0.8		38.0	0.118	7.5	17.7	44.7	0.131	7.1	8.1	44.0	0.114	9.0	7.6	34.4	0.144	10.2	6.1
Range	20–22		116.6–250.0	0.017–0.399	46–71	40–112	108.5–260.9	0.014–0.547	49–72	37–67	109.4–279.5	0.013–0.324	48–79	39–63	72.4–206.8	0.014–0.401	49–87	33–59
Skewness	0.00		0.37	1.81	0.45	1.51	0.45	3.19	0.23	0.12	0.92	1.16	0.97	0.42	0.23	1.22	1.23	-0.22
Kurtosis	-1.62		-0.69	1.99	-0.84	2.62	-0.67	10.90	-1.02	-0.53	0.81	-0.57	0.29	-0.91	0.14	-0.36	1.50	1.01
**Group B**			SR(-)				SR(+)				SR(+)				SR(-)			
No.	Age (years)	Sex	DDT	Steepness	BBT	NHPT	DDT	Steepness	BBT	NHPT	DDT	Steepness	BBT	NHPT	DDT	Steepness	BBT	NHPT
16	20	male	215.4	0.071	48	48	175.3	0.065	47	46	151.2	0.069	52	47	165.2	0.070	51	49
17	20	female	202.1	0.037	57	75	183.5	0.085	58	59	183.5	0.085	59	51	197.9	0.037	64	56
18	20	male	144.1	0.052	57	36	100.2	0.031	58	36	74.8	0.177	64	37	140.9	0.039	65	34
19	20	female	112.1	0.036	54	54	91.6	0.065	62	74	68.1	0.357	63	44	95.0	0.048	63	54
20	20	male	158.3	0.064	52	66	141.7	0.064	56	45	117.5	0.072	54	46	101.6	0.253	56	47
21	21	female	191.6	0.065	50	48	121.9	0.037	55	38	119.7	0.028	59	45	126.3	0.047	59	40
22	21	male	216.4	0.520	59	60	191.8	0.024	67	45	185.0	0.066	67	42	190.6	0.139	69	30
23	21	female	58.6	0.163	55	42	61.2	0.045	62	44	115.4	0.071	64	36	116.5	0.085	66	34
24	21	male	216.6	0.049	55	41	131.3	0.071	53	46	134.4	0.281	55	43	134.4	0.281	54	47
25	21	female	173.9	0.044	41	59	154.5	0.039	40	56	150.0	0.046	41	41	164.7	0.037	44	77
26	22	male	150.0	0.048	58	58	121.0	0.039	62	38	110.6	0.053	70	41	141.7	0.064	72	34
27	22	female	216.5	0.047	63	67	186.6	0.028	60	60	164.3	0.040	56	60	174.2	0.043	63	55
28	22	male	241.7	0.064	45	75	203.6	0.386	46	84	171.9	0.048	50	51	182.4	0.068	48	54
29	22	female	222.9	0.014	60	62	191.2	0.014	57	61	98.6	0.008	65	32	63.8	0.015	60	42
30	22	male	138.3	0.261	58	66	105.0	0.048	63	60	73.7	0.043	60	55	97.7	0.037	63	42
Mean	21.0		177.2	0.102	54.1	57.1	144.0	0.069	56.4	52.8	127.9	0.096	58.6	44.7	139.5	0.084	59.8	46.3
Standard deviation	0.8		48.4	0.126	5.8	11.7	42.2	0.087	7.1	13.3	37.9	0.095	7.3	7.1	38.3	0.077	7.6	11.6
Range	20–22		58.6–241.7	0.014–0.520	41–63	36–75	61.2–203.6	0.014–0.386	40–67	36–84	68.1–185.0	0.008–0.357	41–70	32–60	63.8–197.9	0.015–0.281	44–72	30–77
Skewness	0.00		-0.95	2.75	-0.83	-0.22	-0.28	3.55	-0.92	0.88	-0.06	1.94	-0.74	0.37	-0.24	1.89	-0.58	0.98
Kurtosis	-1.62		0.61	7.91	0.28	-0.88	-1.04	13.23	0.46	0.37	-1.14	3.06	0.71	0.12	-0.82	2.65	-0.26	1.68

SR (+), stochastic resonance on-condition; SR (-), stochastic resonance off-condition; DDT, the delay detection threshold for the delayed visual feedback detection task; Steepness, the steepness of the delay detection probability curve in the delayed visual feedback detection task; BBT, box and block test; NHPT, nine hole peg test.

**Fig 3:**
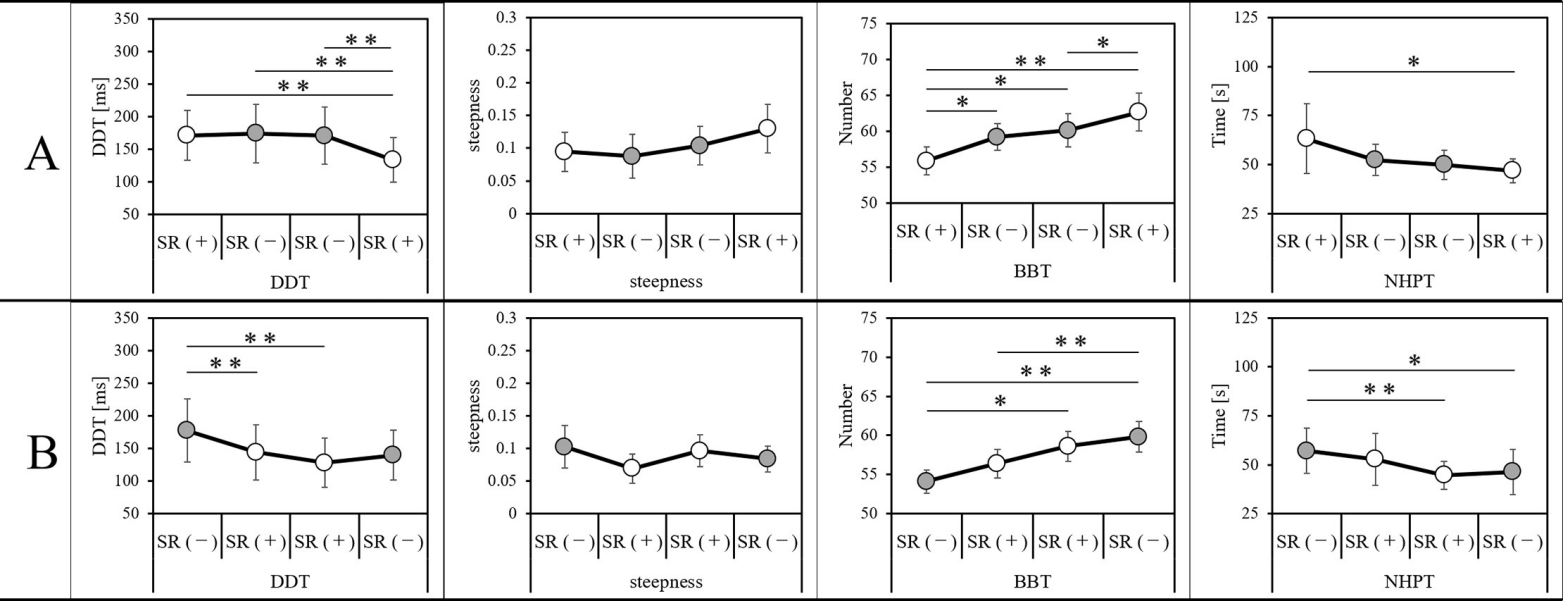
Time course of experimental data and comparisons. DDT, the delay detection threshold for the delayed visual feedback detection task; steepness, the steepness of the delay detection probability curve in the delayed visual feedback detection task; BBT, box and block test; NHPT, nine hole peg test; SR (+), stochastic resonance on-condition; SR (-), stochastic resonance off-condition. White circles, SR on-condition; gray circles, SR off-condition. **P < 0.01; *P < 0.05; Bonferroni-corrected. (A) Time course of the experimental data of 15 participants (Group A; mean age ± standard deviation, 21.0 ± 0.8 years; range, 20–22 years; 8 male participants) who conducted the tasks in the order of SR on-condition, off-condition, off-condition, and on-condition. Error bars represent the standard error or the standard deviation of the mean. (B) Time course of the experimental data of another 15 participants (Group B; 21.0 ± 0.8 years; range, 20–22 years; 8 male participants) who conducted the tasks in the order of SR off-condition, on-condition, on-condition, and off-condition. Error bars represent the standard error or the standard deviation of the mean.

Fig 3 shows the time course and comparison of the DDT, steepness, BBT scores, and NHPT scores in the four blocks.

There was a significant main effect of the DDT in group A (F = 12.522, P < 0.001) as measured by one-way repeated measures ANOVA of block factors. Using multiple comparisons, in group A, the DDT of Block-4 (SR on) was significantly shortened compared with Block-1 (SR on), Block-2 (SR off), and Block-3 (SR off) (vs. Block-1, t = 5.799, P < 0.001; vs. Block-2, t = 5.581, P < 0.001; vs. Block-3, t = 4.732, P = 0.002; all Bonferroni-corrected) (Fig 3A). There was a significant main effect of the DDT in group B (F = 10.854, P = 0.001) as measured by one-way repeated measures ANOVA of block factors. Multiple comparisons in group B showed that the DDT was significantly extended in Block-1 (SR off) compared with Block-2 (SR on) and Block-3 (SR on) (vs. Block-2, t = 5.990, P < 0.001; vs. Block-3, t = 4.833, P = 0.002; all Bonferroni-corrected) (Fig 3B).

The results for the Friedman test of the block factor steepness of groups A and B showed there was no significant main effect (group A, P = 0.345; group B, P = 0.285) (Fig 3A and 3B).

The results for the Friedman test of the block factor of the BBT scores of group A showed a significant main effect (P = 0.001). The results for multiple comparisons showed that, in group A, the BBT scores of Block-1 (SR on) were decreased significantly as compared with Block-2 (SR off), Block-3 (SR off), and Block-4 (SR on) (vs. Block-2, z = -2.998, P = 0.016; vs. Block-3, z = -2.768, P = 0.034; vs. Block-4, z = -3.204, p = 0.008; all Bonferroni-corrected). In addition, the BBT scores for Block-4 (SR on) were increased significantly compared to those of Block-3 (SR off) (z = -2.689, P = 0.043, Bonferroni-corrected) (Fig 3A). There was also a significant main effect shown by one-way repeated measures ANOVA of the block factor results of the BBT scores in group B (F = 12.849, P < 0.001). Multiple comparisons of the BBT scores of group B indicated that they were decreased significantly in Block-1 (SR off) compared to Block-3 (SR on) and Block-4 (SR off) (vs. Block-3, t = -3.575, P = 0.018; vs. Block-4, t = -4.913, P = 0.001; all Bonferroni-corrected). In addition, the BBT scores of Block-4 (SR off) were increased significantly compared to those of Block-2 (SR on) (t = -4.841, P = 0.002, Bonferroni-corrected) (Fig 3B).

Friedman tests of the block factor of the NHPT times in group A showed that there was a significant effect (P = 0.002). Multiple comparison tests in group A showed that the NHPT times of Block-4 (SR on) were significantly shortened compared with those of Block-1 (SR on) (z = -2.756, P = 0.035, Bonferroni-corrected) (Fig 3A). There was a significant effect as demonstrated by one-way repeated measures ANOVA of the block factor of the NHPT times in group B (F = 6.919, P = 0.001). Multiple comparison tests in group B showed that the NHPT times of Block-1 (SR off) were significantly extended compared with those of Block-3 (SR on) and Block-4 (SR of) (vs. Block-3, t = 4.796, P = 0.002; vs. Block-4, t = 3.129, P = 0.044; all Bonferroni-corrected) (Fig 3B).

**Fig 4:**
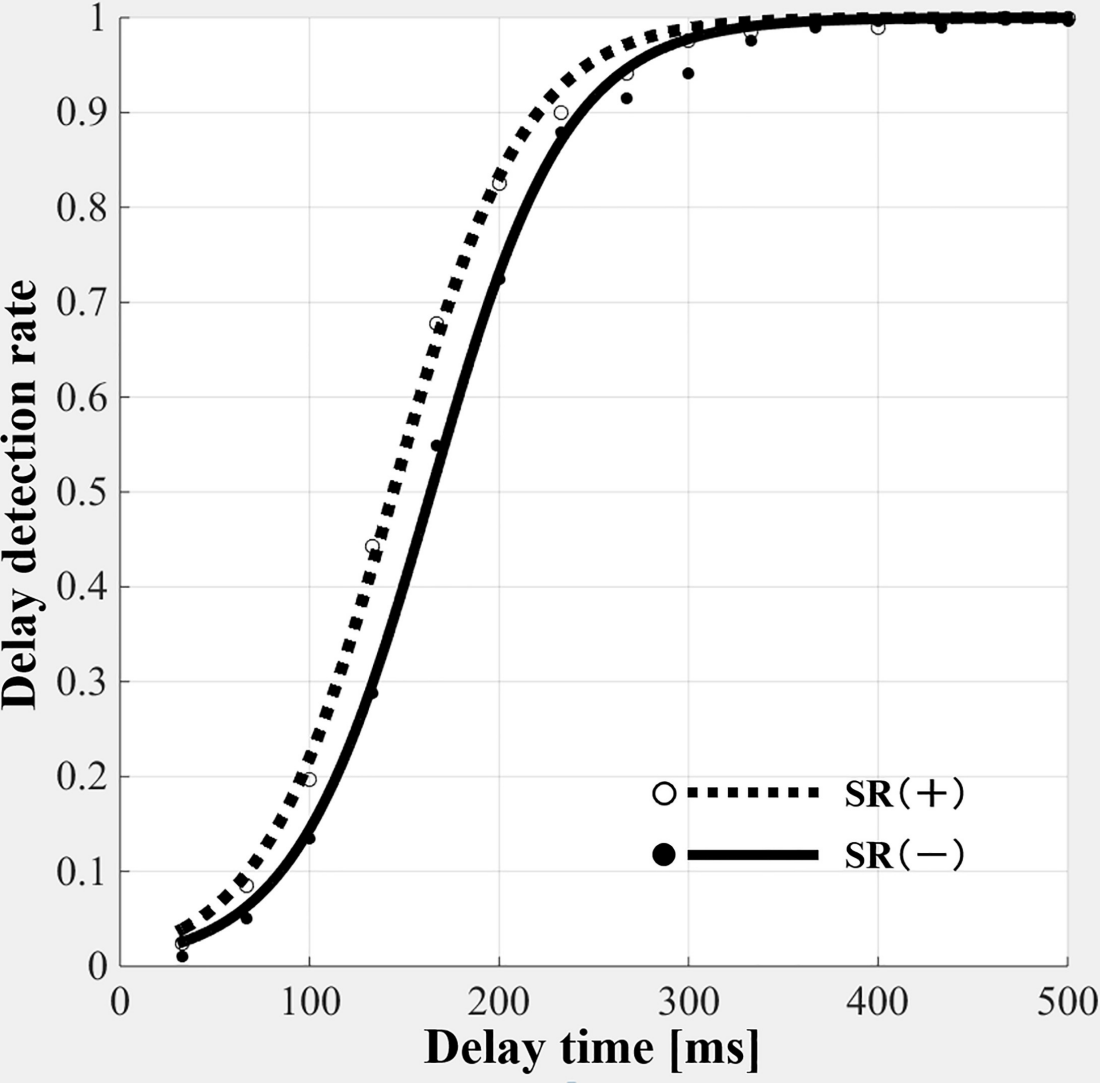
Delay detection probability curves of the delayed visual feedback detection tasks in the stochastic resonance on- and off-conditions. SR (+), stochastic resonance on-condition. SR (-), stochastic resonance off-condition. In delay detection probability curves, shifting the curve to the left and increasing the slope of the curve represent a high performance of visuomotor temporal integration. The probability curve of SR (+) shifted to the left, although its steepness was equivalent compared to the probability curve of SR (-).

Fig 4 shows the delay detection probability curves of the SR on- and off-conditions in the delayed visual feedback detection task.

In delay detection probability curves, shifting the curve to the left shortens the DDT (delay time, ms) and increases the slope of the curve, representing the high performance of visuomotor temporal integration. The probability curve of the SR on-condition moved to the left, although its steepness was equivalent compared to the probability curve of the off-condition.

**Fig 5:**
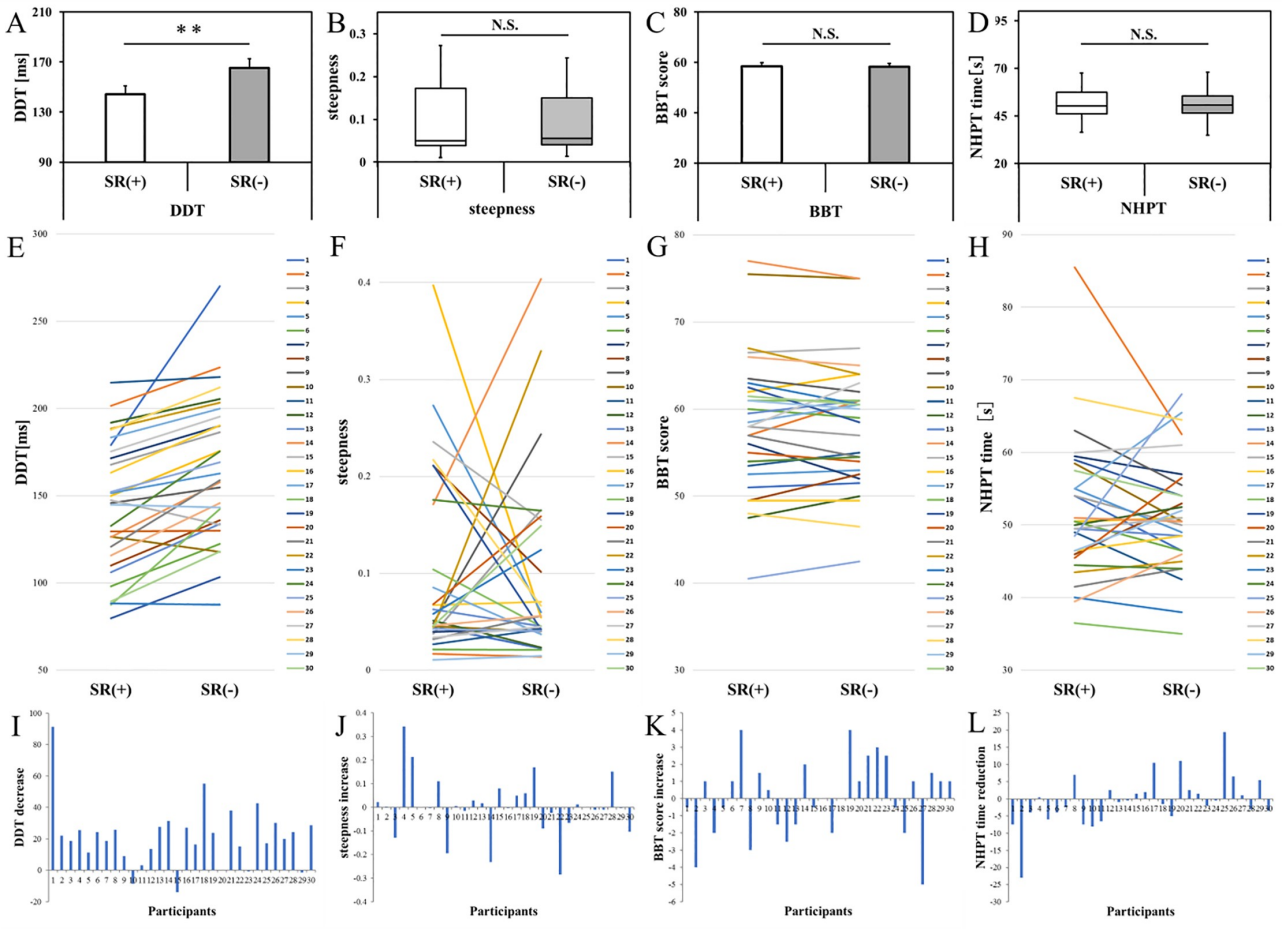
Comparison of the experimental task data in the stochastic resonance on- and off-conditions. DDT, delay detection threshold for the delayed visual feedback detection task; steepness, steepness of the delay detection probability curve in the delayed visual feedback detection task; BBT, box and block test; NHPT, nine hole peg test; SR (+), stochastic resonance on-condition; SR (-), stochastic resonance off-condition. **P < 0.01; *P < 0.05. (A) Comparisons of the DDTs of the SR on- and off-conditions. Error bars represent the standard deviation of the mean. The DDT of the SR on-condition was significantly shortened compared with that of the off-condition (t = -5.840, P < 0.001). (B) Comparisons of the steepness of the SR on- and off-conditions. Lines represent the range of the minimum and maximum. Boxes represent the lower, median, and upper quartiles. There was no significant difference between the SR on- and off-conditions (z = -0.195, P = 0.845). (C) Comparison of the BBT scores of the SR on- and off-conditions. Error bars represent the standard deviation of the mean. There was no significant difference between the SR on- and off-conditions (t = 0.165, P = 0.870). (D) Comparison of the NHPT scores of the SR on- and off-conditions. The error bars represent the range of the minimum and maximum. The boxes represent the lower, median, and upper quartiles. There was no significant difference between the SR on- and off-conditions (z = -0.638, P = 0.524). Individual variability of the DDT (E), steepness (F), BBT scores (G), and NHPT scores (H) between the SR on- and off-conditions. Individual variation of the DDT (I), steepness (J), BBT scores (K), and NHPT scores (L) between the SR on- and off-conditions.

Fig 5A–5D shows the results of comparisons of the DDT, steepness, BBT scores, and NHPT scores under the SR on- and off-conditions.

The DDT under the SR on-condition was significantly shortened compared with the DDT under the SR off-condition (t = -5.840, P < 0.001, effect size, d = 0.48) (Fig 5A). The DDT in the SR on-condition was reduced by an average of 21.2 ms in comparison to the DDT under the SR off-condition. There was no significant difference in steepness between the SR on- and off-conditions (z = -0.195, P = 0.845, effect size, r = -0.04) (Fig 5B). There was also no significant difference in the BBT scores between the SR on- and off-conditions (t = 0.165, P = 0.870, effect size, d = 0.01) (Fig 5C). There was no significant difference in the NHPT scores between the SR on- and off-conditions (z = -0.638, P = 0.524, effect size, r = -0.07) (Fig 5D).

Fig 5E–5H shows the individual variability of the measured values between the SR on- and off-conditions. The individual variability of the DDT (Fig 5E) tended to be shortened from the SR off-condition toward the SR on-condition. However, the individual variability of steepness (Fig 5F) and the BBT and NHPT scores (Fig 5G and 5H) were inconsistent. Fig 5I–5L shows the individual variation (differences) of measurements between the SR on- and off-conditions. Most subjects showed a shortening of the DDT from the SR off-condition to the SR on-condition (Fig 5I). However, individual variation in steepness (Fig 5J) and the BBT and NHPT scores (Fig 5K and 5L) was inconsistent.

## Discussion

In this study, we investigated whether SR improved the delay detection function and/or hand motor function test scores under delayed visual feedback. Multiple comparisons of the measurements between test blocks showed that the DDT did not shorten as the number of trials increased, and SR contributed to the shortening of the DDT. In particular, in group B, the DDT of the SR on-condition (Block-2 and Block-3) was significantly shorter than that of the SR off-condition (Block-1), but the DDT of Block-4 (SR off-condition) was not shortened. Regarding steepness, neither changes due to the number of trials nor changes due to SR were observed. However, hand motor function under delayed visual feedback benefited from the number of trials regardless of whether SR was present or absent. Multiple comparison analyses showed improvements in hand movement performance due to an increase in the number of trials.

In the current study, in order to offset the influence of the number of trials, two designs, SR on-off-off-on and SR off-on-on-off, were used and comparisons were based on the average values. The results showed that SR significantly shortened the DDT, which is an indicator of visuomotor temporal integration, and its effect size was moderate. However, the results also showed that SR did not improve hand motor function under delayed visual feedback.

### Effectiveness of SR for visuomotor temporal integration

In the current study, SR significantly shortened the DDT during delayed visual feedback detection. In general, vibrations can enhance sensory sensitivity by directly stimulating peripheral sensory receptors [2, 19, 26, 41, 85–89]. In the delayed visual feedback detection task, the participants had to compare real-time sensorimotor information with delayed visual feedback. Therefore, SR increased the sensitivity of peripheral sensory receptors and there is a possibility that the error between real-time sensorimotor information and delayed visual feedback became clear. However, in this study, vibrotactile noise was applied to the wrist and a delayed visual feedback detection task was performed with hand opening and closing movements. In other words, the vibrotactile noise-stimulated location (right wrist) and the effect-producing location (right hand) were different. Therefore, there was a possibility that vibrotactile noise stimulation of the wrist did not reach the sensory receptors of the hand. In fact, Kurita et al. [41] reported that mechanical vibrations may lose 90% of its original power when it travels 1–2 cm on the skin. Manfredi et al. [90] reported that mechanical vibrations lose approximately 99% of their power over a distance of 6 cm due to the viscoelastic properties of the skin. Lakshminarayanan et al. [91] assigned vibrotactile noise to four locations (dorsal hand just proximal to the second knuckle, thenar eminence, hypothenar region, and volar wrist) and investigated its influence on fingertip sensation for the distance between the location to which SR was given and the location where the effect occurred. As a result, fingertip sensation was improved by vibrations at 60% of the sensory threshold in all four locations [91]. Similarly, an improvement of tactile sensation at the fingertip by remote subthreshold vibrotactile noise has also been demonstrated in stroke survivors [25]. That is, manipulating the distance between vibration location and effect location does not influence the results [25, 91, 92]. These previous studies suggested that SR might act on the central nervous system, as the same effect was produced even when the distance between SR location and effect location was different.

Vibrotactile noise has been shown to result not only in the increased sensitivity of peripheral sensory receptors but also in increased cortical and spinal neuronal activity in humans [93, 94]. In addition, vibrotactile noise increases the synchronization of neuronal firing between the spinal cord and sensorimotor cortex and between different brain areas [29, 93–96]. This increased neural synchronization can facilitate neural communication for perception between spinal and cortical levels [96, 97]. In the current study, we did not measure central nervous system activity, so this idea is completely speculative, but the observed improvement of visuomotor temporal integration by SR was possibly caused by increased spinal and sensorimotor cortical activity and increased neural synchronization.

Seo et al. [98] provided a more direct explanation. They investigated the influence of imperceptible vibrotactile noise on the wrist, as used in the current study, on somatosensory evoked potential of fingertip touch [98]. As a result, the peak-to-peak somatosensory evoked potentials of the sensorimotor cortex were significantly increased by imperceptible vibrotactile noise (SR on) compared to the absence of vibrotactile noise (SR off) [98]. In addition, this increase spreads not only to the somatosensory cortex and motor cortex but also to the premotor cortex and posterior parietal cortex [98]. Previous studies investigating brain regions involved in delayed visual feedback detection (visuomotor temporal integration) have consistently demonstrated that the parietal cortex and premotor cortex are important regions for this process [76, 99–112]. Nobusako et al. [47] showed that the distortion of visuomotor temporal integration was derived from damage to the premotor cortex and parietal cortex, using voxel-based lesion symptom mapping analyses. Therefore, the improvement of visuomotor temporal integration by SR shown in the current study may have been caused by the activation of the cerebral cortex region responsible for visuomotor temporal integration.

### SR does not improve hand motor function under delayed visual feedback

Unlike previous studies [31, 33, 37, 40–42, 74], SR did not improve hand motor function under delayed visual feedback in the current study.

The cause of these results may be that the effect of motor learning by repeated trials exceeded the effects of SR treatment. This explanation was obvious from the temporal changes and the comparison results shown in Fig 3. The BBT and NHPT scores increased and decreased, respectively, as the number of trials increased. Another possible explanation is that the disturbance effect on sensory-motor integration, that is, the negative effect of delayed visual feedback (267 ms) on self-generated movements, may have exceeded the improvement effect (reduced by 21.2 ms in the delayed visual feedback detection tasks) observed after SR treatment. Therefore, as several studies have shown [31, 33, 37, 40–42, 74], SR may improve hand motor function under real-time conditions. However, as demonstrated in the current study, SR may not have a positive effect on motor function in the presence of large distortions of visuomotor temporal integration.

### Limitations of the current study

It is important to note that there were several limitations in this study. We showed that SR improved visuomotor temporal integration as measured by delayed visual feedback detection tasks. However, we did not measure somatosensory susceptibility or central nervous system activity. Therefore, the reason why SR improved visuomotor temporal integration remains unclear. Further studies require measurements of somatosensory susceptibility and central nervous system activity using imaging techniques.

In order to investigate the influence of SR on motor function, we used two standard hand motor function tests under delayed visual feedback. As a result, it was shown that SR did not affect the hand motor tasks under a delayed visual feedback time of 267 ms. However, there is still the possibility that the influence of SR is due to more detailed kinetics/kinematics indices such as motion trajectory and grip force. Therefore, future studies should include measurements of more detailed hand motor functions using grip/load force devices and three-dimensional motion analysis systems.

The participants in the study were healthy young adults. Therefore, because they already had healthy/normal motor function, there is a possibility that SR did not have an effect. SR may only have the potential to be effective for subjects with impaired motor function.

In the current study, we used two block designs of SR on-off-off-on and SR off-on-on-off to minimize the effects of the number of trials. However, we did not test other block designs such as SR on-off-on-off and SR off-on-off-on. Therefore, in order to determine fully the effects of SR, it will be important to verify our results by using a different design from that used in the current study. In addition, it may be a better strategy to examine the effects of SR after practicing the BBT and NHPT procedures in order to reach a performance plateau before the experimental tests are performed.

In this study, the intensity of vibrotactile noise was set to 60% of the sensory threshold. This was based on previous results [25, 42, 74, 91], but other intensities should also be considered.

The current study was a preliminary study in young healthy right-handed subjects to assess the necessary parameters for using SR in patients with deficits in visuomotor temporal integration such as developmental coordination disorders and limb-apraxia. Therefore, future studies are also needed to investigate the effects of SR on subjects of other age ranges and those who are left handed.

Future studies that address these limitations are necessary to understand better the usefulness of SR in treating individuals with deficits in visuomotor temporal integration.

### Future directions

Several previous studies have reported that children with a developmental coordination disorder and adults with limb-apraxia have deficits in visuomotor temporal integration [45–47]. The current study showed that SR improved visuomotor temporal integration; therefore, SR may improve movement disorders in subjects with deficits in visuomotor temporal integration by improving these deficits. However, SR may not be useful if the distortion of the time window for integrating self-generated movements and visual information is very large. This was suggested by the present results that SR did not improve hand motor function under a delayed visual feedback of 267 ms. Therefore, it is necessary to conduct further studies to determine if SR improves movement disorders with deficits in visuomotor temporal integration.

## Conclusions

The current study showed that SR improved visuomotor temporal integration in healthy young adults. However, SR did not contribute to the improvement of self-generated hand motor function under a visual feedback delay of 267 ms. The present study suggested that SR could improve deficits in visuomotor temporal integration. However, this study also suggested that SR may not be useful if the deficits in visuomotor temporal integration are severe. Future intervention studies using SR for movement disorders with deficits in visuomotor temporal integration are necessary to verify these possibilities.
